# Effects of Hydrogen Sulfide at Normal Body Temperature and in the Cold on Isolated Tail and Carotid Arteries from Rats and TRPA1 Knockout and Wild-Type Mice

**DOI:** 10.3390/biomedicines12122874

**Published:** 2024-12-18

**Authors:** Leonardo Kelava, Eszter Pakai, Kazushi Ogasawara, Kata Fekete, Gabor Pozsgai, Erika Pinter, Andras Garami

**Affiliations:** 1 Department of Thermophysiology, Institute for Translational Medicine, Medical School, University of Pecs, 7624 Pecs, Hungary; leonardo.kelava@aok.pte.hu (L.K.); eszter.pakai@aok.pte.hu (E.P.); kazu115oga@gmail.com (K.O.); kata.fekete@aok.pte.hu (K.F.); 2 Department of Pharmacology, Faculty of Pharmacy, University of Pecs, 7624 Pecs, Hungary; gabor.pozsgai@aok.pte.hu; 3 Department of Pharmacology and Pharmacotherapy, Medical School, University of Pecs, 7624 Pecs, Hungary; erika.pinter@aok.pte.hu

**Keywords:** hydrogen sulfide, wire myography, vasomotor response, tail artery, carotid artery, rat, mouse

## Abstract

**Background:** Hydrogen sulfide (H_2_S) is a gasotransmitter that modulates vascular tone, causing either vasodilation or vasoconstriction depending on the vascular bed, species, and experimental conditions. The cold-sensitive transient receptor potential ankyrin-1 (TRPA1) channel mediates H_2_S-induced effects; however, its contribution to the vasomotor responses of different arteries at different temperatures has remained unclear. Here, we aimed to fill this gap by comparing the effects of sodium sulfide (Na_2_S), which is a fast-releasing H_2_S donor, on the isolated carotid and tail skin arteries of rats and mice at cold and normal body temperature with wire myography. Under the same circumstances, we also aimed to compare the effects of the canonical endothelium-dependent and -independent vasodilators, acetylcholine and sodium nitroprusside, respectively. **Methods:** We isolated the carotid and tail arteries from 32 adult Wistar rats and 64 TRPA1 knockout and wild-type mice, and then we studied their vasomotor responses to increasing doses (10^−6^–10^−3^ M) of Na_2_S as well as to acetylcholine and sodium nitroprusside (10^−5^ M for both) at 37 °C and in cold (17 or 20 °C). **Results:** In rat vessels, Na_2_S caused constriction of the carotids and relaxation of the tail arteries, which were not influenced by temperature. In mouse carotids, Na_2_S caused vasorelaxation, which was more pronounced in the cold at a lower dose (10^−4^ M). At a higher dose (10^−3^ M), the dilation was markedly attenuated in the absence of the TRPA1 channel. In the mouse tail arteries, Na_2_S caused vasorelaxation at 37 °C and vasocontraction in the cold. The genetic blockade of TRPA1 channels did not influence the vasomotor responses of the mouse tail arteries. Sodium nitroprusside-induced vasorelaxation was not influenced by any of the investigated factors, while acetylcholine-induced dilation decreased in the cold in all vessel types. **Conclusions:** Our results reveal the function of TRPA1 in the H_2_S-induced dilation of carotid arteries in mice. We also highlight interspecies differences in the vasomotor responses between rats and mice, as well as the importance of the effect of temperature on vascular responses. The implementation of the identified variables in future research can advance our understanding of cardiovascular physiology, especially in conditions with hypothermia (either accidental or therapeutic).

## 1. Introduction

Until the 1980s, hydrogen sulfide (H_2_S) had been viewed mostly as a toxic substance, but, since then, it has been demonstrated that it has important roles in human and animal physiology. Nowadays, it is classified as the third gasotransmitter, along with nitric oxide and carbon monoxide. H_2_S can be synthesized by different enzymes that regulate sulfur metabolism such as cystathionine beta-synthase and cystathionine gamma-lyase, which are involved in trans-sulfuration pathway, as well as 3-mercaptopyruvate sulfurtransferase, which contributes to cysteine degradation. In endothelial cells, H_2_S is mainly produced by cystathionine beta-synthase, and its protective roles in cardiovascular health have been widely studied [1].

As it is challenging to measure H_2_S in tissues because of its small size, volatility, reactivity, and shared reactions between H_2_S probes and other sulfur-containing molecules, its biological roles and exact concentrations in tissues are still being widely discussed. In vitro experiments usually need quantities in the range over 100 µM to produce a biological effect, so this is considered physiological [2]. However, novel methods of detection often establish that the actual concentrations of H_2_S in tissues are in the nanomolar range. Even so, H_2_S concentrations are dynamically regulated, and certain triggers can cause their transient increase, leading to biological responses. The transmitter roles of H_2_S are exerted locally via autocrine and paracrine modes of action [3].

H_2_S can have both systemic and local effects. The inhalation of H_2_S and the central administration of H_2_S donors, such as Na_2_S, into the intracerebroventricular ventricle both cause metabolic responses in mice [4] and rats [5], which consist of decreases in body temperature, mean arterial pressure, heart rate, and oxygen expenditure.

The transient receptor potential ankyrin-1 (TRPA1) is a non-selective cation channel that is the only member of the TRPA subfamily in mammals [6]. TRPA1 channels have gathered a lot of attention due to their involvement in nociception and inflammation but also in many other diseases, including cardiovascular diseases such as hypertension, atherosclerosis, and myocardial infarction [7,8]. In mammals, TRPA1 was originally described as a cold-sensitive channel [9], but it can be activated by a wide spectrum of thermal, physical, and chemical stimuli, such as noxious cold, heat, mechanical stimuli, pungent compounds, and H_2_S [10,11]. In mice, it was shown that TRPA1 was needed for decreases in body temperature and oxygen consumption upon administration of Na_2_S in the central nervous system, but the same effect was not observed with intraperitoneal injection, where only a short-lasting decrease in body temperature occurred. Central Na_2_S injection was accompanied by increased blood flow in the skin, where vasodilation is a common mechanism to elevate heat loss, thereby decreasing deep body temperature [12].

The nature of these systemic responses suggests that H_2_S has an important role in energy balance regulation. H_2_S also causes local effects when added adjacent to vessels, which have been recorded in the literature to cause either vasodilation or vasoconstriction depending on the experimental setup, concentrations, and other parameters, such as oxygen concentration [13], arterial bed, and species [14]. These vasomotor responses might contribute to energy balance regulation in certain conditions through classical thermoregulatory effectors: constriction of skin arteries prevents heat loss in cold, while their dilation in warmth facilitates cooling without water loss [15].

Due to the interactions between H_2_S and thermoregulation, it should be noted that there are underlying differences in thermoregulatory systems of rats and mice, and these should be accounted for in translational research. Mice are often described as facultative heterotherms being able to enter torpor, i.e., a state of regulated hypothermia and decreased oxygen consumption, while rats are typically considered non-torpid animals [16]. Furthermore, due to their increased size, rats resist hypothermia at lower ambient temperatures more effectively than mice.

In humans, hypothermia is defined as a core temperature below 35 °C. It is accidental when the drop in body temperature is unintentional, while in therapeutic hypothermia, the decrease is intentionally induced in order to improve the outcome of diseases (e.g., cardiac arrest, traumatic brain injury). Accidental hypothermia can be primary when the cold defense mechanisms are overwhelmed by the extent of cold exposure, thereby leading to a decrease in the core temperature [17]. It should be noted, however, that the temperature on the surface of the body (e.g., in the skin) can decrease much faster and to a much larger extent from the beginning of the cold exposure [18]. Secondary forms of accidental hypothermia are often associated with an underlying disease, for example, systemic inflammation [19]. In the latter case, spontaneous hypothermia is a distinct, adaptive mechanism to systemic inflammation, which is considered an attempt to increase survival in severe forms of a disease [20]. Interestingly, a cryogenic role for H_2_S signaling in the development of hypothermia associated with severe systemic inflammation has been proposed [21]. In therapeutic hypothermia, the target temperatures can be as low as 20 °C, which can be considered as a neuroprotectant in acute ischemic stroke [22] or severe traumatic brain injury [23]. However, the extent of cooling should be carefully selected in order to achieve the desired effects, which may explain the contradictory results from studies applying different cooling protocols [24]. Adjuvant interventions to improve the benefits of cooling have also been studied, including the combined use of cooling and H_2_S administration, which have led to promising results in experimental animals [25,26,27]. However, how simultaneous cooling and H_2_S administration affects carotid and tail artery functions in rats and mice has not been compared, neither has been the contribution of the cold-sensitive TRPA1 channel studied in these vascular responses.

Since our previous research showed that the intrabrain but not the intraperitoneal administration of Na_2_S can be accompanied by hypothermia [12], we wanted to evaluate if the local application of Na_2_S on tail arteries from mice and rats would also contribute to hypothermia via vasodilation or if it would oppose hypothermia via vasoconstriction. We studied the vasomotor responses of carotid arteries, which are situated closer to the core, and of tail arteries, which are located close to the body surface, to see if H_2_S has distinct roles in thermoregulatory vessels in comparison to conduit vessels. Furthermore, we checked if these local effects in mice are dependent on TRPA1 channels, which were shown to mediate the thermoregulatory actions of H_2_S.

## 2. Materials and Methods

### 2.1. Experimental Animals and Their Housing

The experiments were performed on 32 adult Wistar rats and 64 mice of both sexes. The mice had the gene of the TRPA1 channel homozygously present (wild type, WT; *n* = 32) or absent (knockout, KO; *n* = 32). They were obtained from the Laboratory Animal Centre of the University of Pecs, where they were bred as described in our earlier studies [12,28,29]. All animals were kept in standard plastic cages (model: 1290 D Eurostandard type III; Akronom Ltd., Budapest, Hungary) in a room with an ambient temperature maintained at 21–23 °C and humidity at 30–40%. The room was on a 12/12 h light/dark cycle (lights on at 5:00 a.m.). The animals had access to standard rodent chow and tap water ad libitum. All procedures were conducted under protocols approved by the Institutional Animal Use and Care Committee of the University of Pecs (registration no.: BA02/2000-23/2022, approved on 2 May 2022) and were in accordance with the directives of the National Ethical Council for Animal Research and those of the European Communities Council (86/609/EEC).

### 2.2. Vessel Isolation and Preparation

The mice and rats were anesthetized by an intraperitoneal injection of a ketamine–xylazine cocktail [81.7 (Calypsol; Gedeon Richter Plc., Budapest, Hungary) and 9.3 mg/kg (Sedaxylan; Eurovet Animal Health B.V., Bladel, The Netherlands), respectively]. For isolation of tail arteries, lateral incisions were made, and the skin was removed carefully from the top of the tail. Thin entomological needles were used to separate the tail artery from the connective tissue and long segments of arteries were excised. The preparation and removal of tail arteries were performed under a surgical microscope (model SZX7; Olympus, Tokyo, Japan). Common carotid arteries were ligated and isolated, and then the segments were excised between the ligations. After the removal of the vessels, the animals were euthanized with an intraperitoneal injection of pentobarbital (100 mg/kg).

The excised arteries were placed in Petri dish with gel-covered bottom containing Krebs solution (NaCl: 119 mM, KCl: 4.7 mM, KH_2_PO_4_: 1.2 mM, NaHCO_3_: 25 mM, Mg_2_SO_4_: 1.2 mM, CaCl_2_·2H_2_O: 1.6 mM, EDTA: 0.026 mM, and glucose: 11.1 mM) that was perfused with a mixture of 5% CO_2_ and 95% O_2_. The arteries were dissected into 2 mm long rings, four of which were mounted via tungsten wires on the transducers in the chambers of a wire myograph (DMT 610 M; Wire Myograph Danish Myo Technology, Aarhus, Denmark) prefilled with 5 mL Krebs solution, as in our previous studies [30,31].

### 2.3. Experimental Procedures

With an auxiliary device that we developed recently [31], the water bath of the chambers was maintained at a designated temperature (37 °C, 20 °C, or 17 °C) for at least an hour before the vessels were placed into them. While 37 °C corresponded to normal body temperature, 20 °C and 17 °C were used to mimic cold for the vessels isolated from mice and rats, respectively. Different temperatures for cold in rats and mice were needed because, in contrast with those of the rats, the arteries from mice were unresponsive to the treatments we used to check viability (for details, see below) at 17 °C, but they reliably responded at 20 °C. After insertion into the chambers, the vessels were left to stabilize for 30 min. Before the start of each experiment, normalization was performed according to the manufacturer’s instructions to yield optimal tension of the vessel, and then the vessels were allowed to stabilize for an additional 30 min. Next, the vessels were precontracted with either phenylephrine (10^−5^ M for mice and 10^−4^ M for rats) or 90 mM KCl, and viability tests were performed by checking the responsiveness of the vessels to vasodilators acting on endothelial or vascular smooth muscle cells. Endothelium-dependent vasodilation was evaluated with acetylcholine (10^−5^ M for mice and 10^−4^ M for rats), while endothelium-independent vasodilation was assessed with sodium nitroprusside (SNP; 10^−5^ M for mice and 10^−4^ M for rats).

After the confirmation of the responsiveness of the vessels, the chamber was washed two times with Krebs solution, and then the vessels were precontracted with phenylephrine before the administration of 50 µL of Na_2_S solution (or distilled water as control) to achieve Na_2_S concentrations of 10^−6^, 10^−5^, 10^−4^, and 10^−3^ M in the bath solution of the vessel. In these experiments, phenylephrine was used for precontraction because, in the case of KCl, it could be expected that the potassium ions would influence the Na_2_S-induced responses [32]. At the end of each experiment, the chambers were washed two times with Krebs solution, and then the viability of the vessels was evaluated again.

### 2.4. Drugs and Substances

All drugs and chemicals were purchased from Sigma Aldrich (St. Louis, MO, USA), unless specified otherwise. Na_2_S nonahydrate (Na_2_S·9H_2_O) was used as a donor of H_2_S. It was freshly dissolved and diluted with distilled water on the day of the experiment to 10^−4^–10^−1^ M to deliver the required concentrations (see above) of Na_2_S into the bath solution of the vessels. Phenylephrine hydrochloride, acetylcholine hydrochloride, and SNP were dissolved in distilled water at 10^−5^ M for experiments in mice and 10^−4^ M for experiments in rats.

### 2.5. Statistics

The measurements of each experiment were exported from LabChart 8 (AD Instruments, Dunedin, New Zealand) to Microsoft Excel (version 16.0; Microsoft Corp., Redmond, WA, USA). The analysis was performed in R (version 3.6.1; R Development Core Team, Vienna, Austria). Since H_2_S quickly evaporates from air-bubbled wire myograph chambers [33], the vasomotor response to each concentration of Na_2_S was evaluated until a timepoint just after the reported 200 s halftime of its evaporation. Indeed, it was suggested that the halftime of 10^−4^ H_2_S in myograph chambers containing air-bubbled water solution of 5 mL is 2.46 min [33]. The maximum vasomotor response (constriction or relaxation) within the 200 s time period was determined and divided with the baseline, which was considered as the maximum force obtained during the precontraction of the vessel before administration of the substance of interest. The vessels that did not respond to acetylcholine were excluded from the analysis because of the indication of endothelial dysfunction. The experiments with at least one vessel that complied with the a priori established criteria were included in the analysis, and mean values with standard error (SE) were calculated for each experiment. The values were tested with the Shapiro test for normality. As the relationship between the vasomotor response and Na_2_S concentration was nonlinear, for the mouse data, two-way ANOVA was performed for each concentration with the change in isometric force (i.e., the measure of the vasomotor response) as the dependent variable and genotype and temperature as independent factors. For the data from the rats, one-way ANOVA was used with temperature as an independent factor. Data are presented as the mean ± SE.

## 3. Results

### 3.1. Vasomotor Response to Na_2_S in Carotid and Tail Arteries of Rats and Mice at Different Temperatures

**Figure 1:**
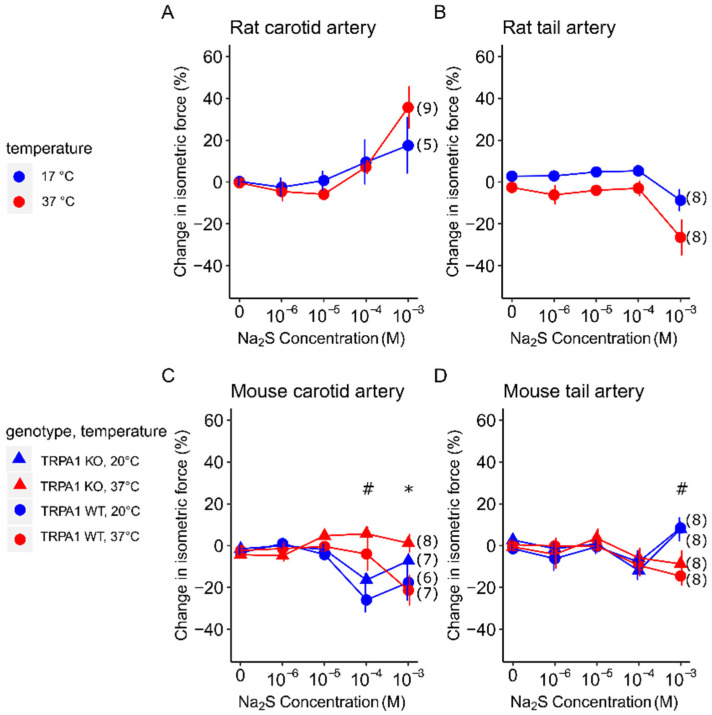
The change in isometric force (expressed as a percentage of the baseline) in response to different concentrations of Na_2_S in (**A**) rat carotid, (**B**) rat tail, (**C**) mouse carotid, and (**D**) mouse tail arteries at normal body temperature (37 °C) and in the cold. Numbers in parentheses indicate the number of animals in each treatment group. ^#^ indicates a statistically significant difference between temperature groups (37 °C vs. cold; *p* < 0.05); * indicates a statistically significant difference between genotypes (TRPA1 KO vs. WT; *p* < 0.05). The baseline isometric force values of the arteries were as follows: rat carotid at 37 °C 1.59 ± 0.31 mN, in cold 0.27 ± 0.12 mN; rat tail at 37 °C 4.01 ± 1.53 mN, in cold 2.41 ± 0.61 mN; TRPA1 WT mouse carotid at 37 °C 1.09 ± 0.20 mN, in cold 0.78 ± 0.21 mN; TRPA1 KO mouse carotid at 37 °C 0.82 ± 0.14 mN, in cold 0.42 ± 0.05 mN; TRPA1 WT mouse tail at 37 °C 1.15 ± 0.29 mN, in cold 0.61 ±0.09 mN; TRPA1 KO mouse tail at 37 °C 0.76 ± 0.17 mN, in cold 0.50 ± 0.40 mN.

Na_2_S caused different vasomotor responses in the range from 10^−4^ to 10^−3^ M, which varied depending on the species, arterial bed, temperature, and genotype (Figure 1).

In the rat carotid arteries at 37 °C, Na_2_S significantly (*p* = 0.009) increased the isometric force at 10^−3^ M compared to distilled water (Figure 1A). At this concentration, the vasoconstriction was somewhat less at 17 °C than at 37 °C, but the difference in the vasomotor response was not statistically significant between the two temperatures (35.7 ± 10.1 vs. 13.2 ± 14.9%).

In the rat tail arteries (Figure 1B), Na_2_S caused a significant (*p* = 0.031) decrease in the isometric force compared to distilled water also at the highest applied concentration of 10^−3^ M. The relaxation of the vessels tended to be more pronounced at 37 °C (−26.5 ± 8.7%) than in the cold (−8.7 ± 5.2%) [*F*_(1,14)_ = 3.27, *p* = 0.0922].

In the mouse carotid arteries, Na_2_S at 10^−4^ M caused the relaxation of the vessels in TRPA1 WT mice, which was more pronounced at 20 °C than at 37 °C (−26.1 ± 6.1 vs. −4.1 ± 8.0%) (Figure 1C). The effect of temperature was significant on the vasodilation response [*F*_(1,1,1,24)_ = 16.606, *p* = 0.000463]. The vasomotor response of the vessels from TRPA1 KO mice was similar to that of the WT mice, without any meaningful difference between the genotypes. At the 10^−3^ M concentration, Na_2_S caused vasodilation in the vessels of the TRPA1 WT mice, which was not meaningfully different between 37 and 20 °C (−21.4 ± 7.2 vs. −17.7 ± 8.7%). Importantly, however, the genotype of the mice had a significant [*F*_(1,1,1,24)_ = 8.263, *p* = 0.00835] effect on the vasomotor response at this concentration. Tukey’s post hoc test revealed that the arteries of the TRPA1 KO mice showed markedly smaller vasomotion at 37 °C (1.1 ± 4.2%; *p* = 0.04864) and a somewhat, though not significantly, smaller response at 20 °C (−7.4 ± 2.6%; *p* = 0.62025) compared to the carotid arteries of the TRPA1 WT mice.

In the mouse tail arteries, only the highest concentration (10^−3^ M) of Na_2_S elicited a vasomotor response [*F*_(1,1,1,28)_ = 12.949, *p* = 0.00122], which occurred in both the TRPA1 WT and KO mice without a significant inter-genotype difference (Figure 1D). In contrast with the genotype, at this concentration of Na_2_S, the temperature had a notable influence on the vasomotor response, since vasodilation developed at 37 °C (−14.9 ± 4.7 and −9.1 ± 6.6%) whereas vasoconstriction occurred at 20 °C (8.1 ± 4.9 and 7.5 ± 5.7%) in both the TRPA1 WT and KO mice.

### 3.2. Acetylcholine-Induced Vasomotor Responses in Carotid and Tail Arteries of Rats and Mice at Different Temperatures

**Figure 2:**
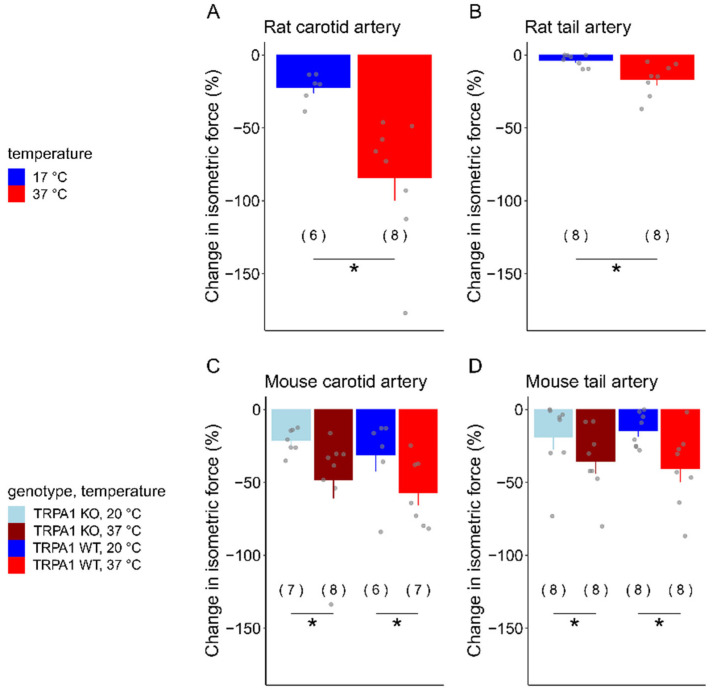
The change in isometric force (expressed as a percentage of the baseline) in response to acetylcholine (10^−4^ M for rats and 10^−5^ M for mice) in (**A**) rat carotid, (**B**) rat tail, (**C**) mouse carotid, and (**D**) mouse tail arteries at normal body temperature (37 °C) and in cold. Numbers in parentheses indicate the number of animals in each treatment group. * indicates a statistically significant difference between temperature groups (37 °C vs. cold; *p* < 0.05). The baseline isometric force values of the arteries were as follows: rat carotid at 37 °C 1.51 ± 0.28 mN, in cold 0.27 ± 0.10 mN; rat tail at 37 °C 3.53 ± 1.04 mN, in cold 2.92 ± 0.73 mN; TRPA1 WT mouse carotid at 37 °C 1.09 ± 0.20 mN, in cold 0.78 ± 0.21 mN; TRPA1 KO mouse carotid at 37 °C 0.82 ± 0.14 mN, in cold 0.42 ± 0.05 mN; TRPA1 WT mouse tail at 37 °C 1.15 ± 0.29 mN, in cold 0.61 ±0.09 mN; TRPA1 KO mouse tail at 37 °C 0.76 ± 0.17 mN, in cold 0.50 ± 0.40 mN.

The effect of temperature on the vasomotor response to acetylcholine was significant in all four arterial beds studied (Figure 2).

In the rat carotid arteries, a significant difference was observed in the acetylcholine-induced decrease in the isometric force at 17 °C (−22.1 ± 4.0%) compared to at 37 °C (−84.3 ± 15.4%) [*F*_(1,12)_ = 11.51; *p* = 0.00535] (Figure 2A). In the rat tail arteries, the extent of vasomotor response was smaller, but, similar to the carotid arteries, the acetylcholine-induced vasorelaxation was significantly more pronounced at 37 °C (−16.6 ± 4.0%) than at 17 °C (−3.7 ± 1.5%) (*F*_(1,14)_ = 9.465; *p* = 0.00821) (Figure 2B).

In the mice carotid arteries, a significant difference was also observed between the two temperatures [*F*_(1,1,1,24)_ = 4.991; *p* = 0.0143); however, we did not find a meaningful inter-genotype difference (Figure 2C). In the TRPA1 WT mice, the extent of vasorelaxation was −56.9 ± 8.8% at 37 °C and −31.2 ± 11.2% at 20 °C, while, in the TRPA1 KO mice, it was −56.3 ± 20.8 and −21.2 ± 3.1%, respectively. Similar to the carotids, in the mouse tail arteries, the effect of temperature was significant on the vasorelaxation response [*F*_(1,1,1,28)_ = 7.297; *p* = 0.0116], but there was no meaningful inter-genotype difference (Figure 2D). In the TRPA1 WT mice, the decrease in isometric force was −40.4 ± 9.3% at 37 °C and −13.3 ± 4.1% at 20 °C, while, in TRPA1 KO mice, it was −35.3 ± 8.3 and −18.7 ± 8.9%, respectively.

### 3.3. SNP-Induced Vasomotor Responses in Carotid and Tail Arteries of Rats and Mice at Different Temperatures

**Figure 3:**
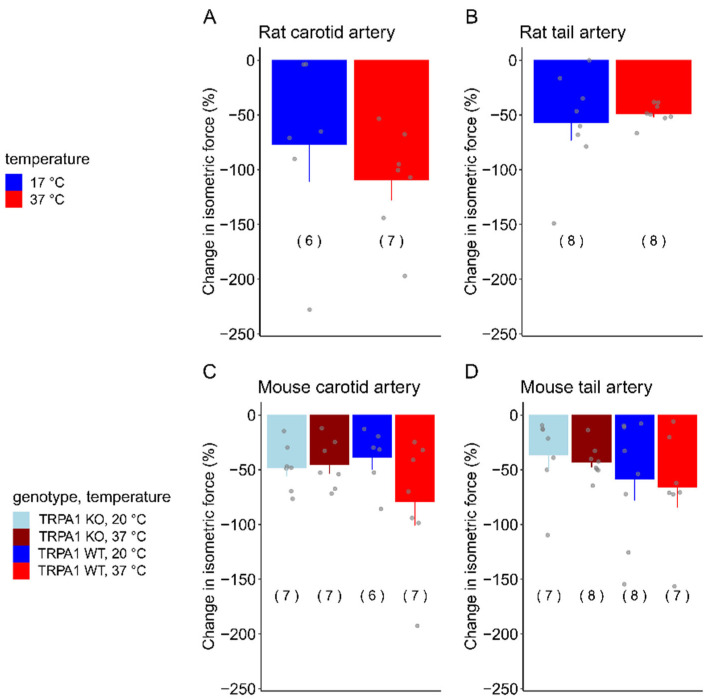
The change in isometric force (expressed as a percentage of the baseline) in response to SNP (10^−4^ M for rats and 10^−5^ M for mice) in (**A**) rat carotid, (**B**) rat tail, (**C**) mouse carotid, and (**D**) mouse tail arteries at normal body temperature (37 °C) and in cold. Numbers in parentheses indicate the number of animals in each treatment group. The baseline isometric force values of the arteries were as follows: rat carotid at 37 °C 1.59 ± 0.31 mN, in cold 0.27 ± 0.12 mN; rat tail at 37 °C 4.01 ± 1.53 mN, in cold 2.41 ± 0.61 mN; TRPA1 WT mouse carotid at 37 °C 1.25 ± 0.31 mN, in cold 0.76 ± 0.17 mN; TRPA1 KO mouse carotid at 37 °C 0.80 ± 0.16 mN, in cold 0.42 ± 0.05 mN; TRPA1 WT mouse tail at 37 °C 1.25 ± 0.31 mN, in cold 0.61 ±0.09 mN; TRPA1 KO mouse tail at 37 °C 0.76 ± 0.17 mN, in cold 0.50 ± 0.40 mN.

In contrast with acetylcholine, temperature did not have a significant effect on the vasomotor response to SNP in any of the studied vascular beds (Figure 3).

In the rat carotid arteries, the SNP-induced vasorelaxation seemed to be less at 17 °C (−76.9 ± 33.6%) than at 37 °C (−109.3 ± 18.3%), but the difference was not statistically significant [*F*_(1,12)_ = 1.6211888; *p* = 0.227] (Figure 3A). In the rat tail arteries, a similar vasorelaxation occurred in response to SNP at 17 °C (−56.7 ± 16.1%) and 37 °C (−48.5 ± 3.3%), again without a significant difference between the two temperatures [*F*_(1,14)_ = 0.249; *p* = 0.626] (Figure 3B).

The vasomotor responses of the mouse arteries to SNP were similar to those that we observed in the rats. The temperature of the bath solution and the genotype of the mice did not have a significant influence on the response in either carotid arteries (Figure 3C) or tail arteries (Figure 3D). In the carotid arteries of the TRPA1 WT mice, the SNP-induced vasorelaxation was −79.0 ± 21.9% at 37 °C and −38.6 ± 10.9% at 20 °C, while in TRPA1 KO mice, it was −45.0 ± 8.5% at 37 °C and −47.7 ± 8.1 at 20 °C (Figure 3C). SNP also caused vasorelaxation in the tail arteries, which was −65.5 ± 18.0% at 37 °C and −58.4 ± 19.7% at 20 °C in the TRPA1 WT mice, while −42.7 ± 5.3% at 37 °C and −89.7 ± 54.5% at 20 °C in the TRPA1 KO mice.

## 4. Discussion

In the present study, we investigated how the vasomotor responses of isolated vessels to different substances (Na_2_S, acetylcholine, and SNP) depend on the species (rat vs. mouse), vascular bed (carotid vs. tail arteries), temperature of the bath solution (body temperature vs. cold), and function of the TRPA1 channel (present vs. absent). We showed that SNP-induced vasorelaxation was not influenced by any of the investigated factors, while acetylcholine-induced dilation was decreased in the cold in all vessel types. The vasomotor response to Na_2_S greatly varied among the different experimental conditions. In the rat vessels, it caused constriction in the carotids but relaxation in the tail arteries, in which the responses were not significantly influenced by the temperature of the bath solution. In the mouse carotids, vasorelaxation occurred in response to Na_2_S, which was more pronounced in the cold at a lower dose (10^−4^ M), but it was not temperature-dependent at a higher dose (10^−3^ M). However, at the higher dose, the dilation was markedly attenuated in the absence of the TRPA1 channel. In the mouse tail arteries, Na_2_S had an effect only at a higher dose (10^−3^ M), which was vasorelaxation at 37 °C and vasocontraction in the cold. The genetic blockade of the TRPA1 channels did not influence the vasomotor responses of the mouse tail arteries.

Our results seemingly contradict previous findings showing that the acetylcholine-induced vasodilation of mouse tail arteries was not inhibited by decreasing the temperature to 28 °C [34]. However, in our study, we used a significantly lower temperature (17–20 °C), which was maintained with our recently developed heat exchanger device [31]. In the same experimental setup, we previously showed in rat tail arteries that, in this temperature range, the KCl-induced contraction is attenuated, which can be partially reversed by increasing the concentration of KCl from 60 mM to 90 mM [31]. We suggested that this attenuation is due to the inhibition of Na^+^/K^+^-ATPase by cold, which was shown by other researchers [35], and can lead to the development of more positive membrane potential, thereby hindering the influx of K^+^ ions into the cells. Since acetylcholine-induced vasodilation is also mediated by small- and intermediate-conductance potassium channels, this can also explain why it was significantly reduced in the cold during our experiments. In agreement, it was shown that the vasodilation induced by acetylcholine is affected by potassium concentration [36]. In contrast with acetylcholine, SNP-induced vasorelaxation was not affected by temperature in our study. Nitric oxide donors, like SNP, induce vascular smooth muscle relaxation mainly through the activation of cyclic GMP-dependent pathways [37], and the lack of involvement of Na^+^/K^+^-ATPase activation in SNP-induced effects was also shown [38], which can explain the difference in the effect of temperature between acetylcholine and SNP. We also showed that the TRPA1 channel did not contribute to the vasomotor response to acetylcholine and SNP in any of the tested experimental conditions.

The effect of Na_2_S was observed at doses of 10^−4^ and 10^−3^ M, which is in accordance with previous findings, suggesting these are the concentrations needed to observe distinct biological effects in vitro [2]. Furthermore, during in vivo experiments involving a closed cranial window in mice, Na_2_S caused vasodilation in dural and pial arteries at a low dose of 8 × 10^−6^ mol/kg [39], which was observed within 1 min. Interestingly, both of these arteries express TRPA1 channels in the endothelium, and the dilation was dependent on the TRPA1 in the dural arteries, while in pial arteries, it was dependent on KCa3.1 channels [39]. In our study, we did not find a significant difference between the vasomotor response of the tail arteries isolated from the TRPA1 KO and WT mice. This is in contrast with two previous in vivo experiments on cutaneous arteries from the ear and paws [40,41]. In one of the studies, the vasodilation induced by a topical H_2_S donor was attenuated in the ear skin arteries of TRPA1 KO mice compared to their WT littermates [40], while in the other study, the cold-induced vasomotor responses triggered by the immersion of the paws in cold water were reduced in TRPA1 KO mice compared to WT controls [41]. The difference between these findings and our results could be due to differences in the studied vascular beds (tail vs. ear/paw) or in the experiment design (in vitro vs. in vivo). The TRPA1 channel is expressed in the vascular system in endothelial cells, e.g., in pial and dural arteries, as well as in perivascular neurons [39,42]. Wire myography is usually used to assess the functions of endothelial cells and vascular smooth muscle cells, but several groups have reported that functional nerve endings might be present in the isolated vessel segments as well [43,44,45]. However, these neurons would be expected to be damaged and equilibrated with the bath solution, which questions how reliably the activation of ion channels on their plasma membrane could stimulate synaptic transmission. Indeed, the presence of neurons in wire myograph bath chambers was demonstrated by using calcium-independent activators of synaptic transmission such as tyramine [44] or electric field stimulation coupled with vesicle transport inhibitors such as guanethidine [43]. The technical difficulties in investigating neurogenic responses with wire myography were also summarized, emphasizing that although neurotransmitters can be stored and released in the nerve endings, they can be also easily depleted [45]. Hence, it is plausible that TRPA1 channels expressed on neural elements contribute to the cold- and H_2_S-induced vasomotor responses in vivo, as shown in previous studies [40,41]; however, in wire myography experiments on isolated arteries, the function of neural TRPA1 channels is probably not so significant. Our study suggests that the TRPA1 channels expressed on non-neural structures of the tail artery do not play a role in the studied vasomotor responses.

The vasomotor responses of carotid arteries were the most varied. In mice, Na_2_S caused vasodilation, whereas it induced vasoconstriction in rats. The reasons underlying these differences might be multifaceted. It was shown that H_2_S also causes vasodilation in the rabbit common carotid [46]; thus, the vasoconstriction observed in the present study might be a specific adaptation in rats, which could promote shunting of the H_2_S-rich blood toward the liver, where three enzymatic pathways enable the oxidation and clearance of sulfide from the blood [47]. An alternative explanation might be associated with differences in the thermoregulatory system between the two species [48]. Mice have the ability to enter torpor, a hypometabolic state where their core temperature, activity, and oxygen consumption drop [49], while, in rats, the existence of torpor is debated [16]. H_2_S was proposed as an important mediator in the activation of this energy-conserving mechanism [50]. During a torpor bout, the peripheral vascular resistance increases; thus, tail arteries would be expected to constrict, while mean arterial pressure drops; thus, conduit arteries, such as carotids, dilate to accommodate for blood that leaves peripheral arteries [49]. We observed similar vasomotor responses to Na_2_S in the cold in the present study: mouse carotid arteries strongly dilated, whereas tail arteries constricted. Furthermore, in mouse carotid arteries, the vasorelaxation induced by 10^−3^ M of Na_2_S was attenuated in TRPA1 KO mice, suggesting that the H_2_S-induced activation of TRPA1 channels contributes to this vasomotor response. It is an interesting finding that the role of TRPA1 was more pronounced at 37 °C than in the cold in our experiments. It should be mentioned, however, that previous studies showed that, during cooling, the TRPA1 channel can be desensitized [51,52]; thus, the loss of its function due to cooling-induced desensitization in the TRPA1 WT mice may explain why there was no significant difference in the vasomotor response of the carotid arteries to H_2_S between the TRPA1 WT and KO mice. As an alternative explanation, it was also demonstrated that TRPA1 plays a critical role in the physiological response to noxious heat in rodents [53]. The role of TRPA1 as a heat sensor in mammals was also supported by other studies [54,55]. It was concluded that TRPA1 possesses a U-shaped thermosensitivity in mammals, which enables the channel to participate in sensing warmth in addition to noxious cold [54]. Therefore, it is possible that the heat sensor function is required for the vasomotor response to H_2_S, but its cold sensor function is not essential. In support of this hypothesis, we previously showed that TRPA1 does not function as a cold sensor for thermoregulation in rats and mice [28].

Interestingly, in 2002, Mustafa et al. observed cold-induced vasodilation in carotid arteries from rabbits [56]. In their review two decades later, they reported the same effect in the carotid artery, aorta, and jugular vein in several animal models, including rats. They concluded that this response was not endothelium-dependent, was not neurogenic or myogenic, did not involve nitric oxide or carbon monoxide, and did it involve any vasodilator compound released from vessels, as the addition of more vessel segments to the chambers did not increase the response. The authors proposed that this response is mediated by unknown thermal receptors in the vascular smooth muscle cells [57]. Hyperpolarization and subsequent vasodilation were indeed shown to be mediated through calcium entry via TRP channels in multiple vascular beds [58].

Limitations of our study should be also mentioned. It would have been advantageous to study arterioles instead of the larger arteries, but, unfortunately, our experimental setup did not allow us to mount vessels with such small diameter in the wire myograph. The function of arterioles, rather than larger arteries, is usually involved in physiological reactions; thus, more pronounced results could have been obtained with arterioles instead of arteries. Nevertheless, our findings can be of importance, since artery functions significantly influence arterioles through structural adaptations, regulatory mechanisms involving neural and hormonal control, and local metabolic responses. These interactions ensure that arterioles can effectively manage blood pressure and flow, adapting dynamically to the needs of different tissues throughout the body. Understanding these relationships is vital for comprehending the cardiovascular health and disease processes affecting the vascular system [59].

As a further limitation of our study, it should be also mentioned that although we aimed to study the Na_2_S-induced vasomotor responses as potential mechanisms of hypothermia, an in vivo study could provide more direct and accurate information about whether the observed vasomotor changes play a role in the mediation or prevention of hypothermia. Incorporating in vivo studies along with hemodynamic measurements could also better elucidate the effects of the used substances on vascular dilation, contraction, and blood flow. We should also note that, at this point, it is unclear whether the vasomotor response of human arteries to H_2_S would resemble those of rat or mouse vessels (or perhaps none of them) due to the lack of comparative physiological experiments among the three species. Nevertheless, our present findings call attention to important interspecies differences in the vascular biology of commonly used preclinical models, which should be considered in future research, especially when taking into account their translational research value.

It is important to mention that the combination of therapeutic hypothermia and H_2_S administration was proposed as a novel approach for patients with certain ischemic conditions [25] since the combined treatment resulted in superior outcomes compared to either treatment alone in experimental models of cardiac arrest and cerebral ischemia [25,26,27]. Our results can help to better understand the vascular effects of the combined use of cold and H_2_S by showing, for the first time to the best of our knowledge, their combined effects on mouse and rat arteries.

## 5. Conclusions

In conclusion, in response to Na_2_S in rat carotid arteries, we observed vasoconstriction, whereas, in tail artery vasodilation, which responses were not influenced by temperature. In mice carotids, vasorelaxation occurred, which was more pronounced in the cold at lower doses and mediated by the TRPA1 channel at higher doses. In the mouse tail arteries, vasodilation developed in the warmth but constriction in the cold without the TRPA1 channel influencing the response. The TRPA1 channels did not play a role in the acetylcholine- and SNP-induced vasorelaxation, which suggests a specific function for the TRPA1 channel in the H_2_S-induced relaxation of carotid arteries. Our research emphasizes important differences between the vasomotor responses of rats and mice, central and peripheral arteries, as well as between experiments conducted at normal body temperature and in the cold.

## Data Availability

Data are contained within this article.
